# Identifying Themes for Assessing Cancer-Related Cognitive Impairment: Topic Modeling and Qualitative Content Analysis of Public Online Comments

**DOI:** 10.2196/34828

**Published:** 2022-05-25

**Authors:** Shelli R Kesler, Ashley M Henneghan, Whitney Thurman, Vikram Rao

**Affiliations:** 1 School of Nursing University of Texas at Austin Austin, TX United States

**Keywords:** cognitive, natural language processing, cancer, oncology

## Abstract

**Background:**

Cancer-related cognitive impairment (CRCI) is a common and significant adverse effect of cancer and its therapies. However, its definition and assessment remain difficult due to limitations of currently available measurement tools.

**Objective:**

This study aims to evaluate qualitative themes related to the cognitive effects of cancer to help guide development of assessments that are more specific than what is currently available.

**Methods:**

We applied topic modeling and inductive qualitative content analysis to 145 public online comments related to cognitive effects of cancer.

**Results:**

Topic modeling revealed 2 latent topics that we interpreted as representing internal and external factors related to cognitive effects. These findings lead us to hypothesize regarding the potential contribution of locus of control to CRCI. Content analysis suggested several major themes including symptoms, emotional/psychological impacts, coping, “chemobrain” is real, change over time, and function. There was some conceptual overlap between the 2 methods regarding internal and external factors related to patient experiences of cognitive effects.

**Conclusions:**

Our findings indicate that coping mechanisms and locus of control may be important themes to include in assessments of CRCI. Future directions in this field include prospective acquisition of free-text responses to guide development of assessments that are more sensitive and specific to cognitive function in patients with cancer.

## Introduction

A condition known colloquially as *chemobrain*, cancer-related cognitive impairment (CRCI) affects an estimated 60% or more of patients with cancer [1,2]. CRCI is an interesting illustration of the significant effects that systemic disease or its therapies can have on brain function. Cognitive deficits decrease quality of life in patients with cancer and are independent predictors of survival [3-5]; however, assessment of CRCI remains challenging. Specifically, despite the significant and widespread brain changes observed in neuroimaging studies of CRCI, behavioral assessments show less consistent effects [6-8].

Objectively, CRCI is primarily assessed using standardized neuropsychological testing. However, these measures tend to have poor ecological validity [9-11] and may lack adequate sensitivity and specificity for CRCI [12]. Self-report measures tend to be more sensitive to CRCI, but have their own set of disadvantages in terms of administration and interpretation [13] and also do not always detect CRCI [14]. Most existing assessments are not cancer specific, and therefore, they may not sufficiently include themes or domains that are important to cancer survivors. Qualitative research is best suited for uncovering such themes but has been limited to date in this field.

The aim of this study was to elucidate qualitative themes surrounding the cognitive effects of cancer to better inform development of cancer-specific self-report assessments. We employed 2 text analysis approaches: topic modeling and traditional content analysis. Both methods use unstructured, free-text responses to assess symptoms and functioning. Topic modeling is a text mining technique that seeks to interpret the rich data inherent in written language using machine learning algorithms to identify important themes, or topics [15]. This method removes some of the labor-intensive aspects of traditional qualitative analysis by automatically quantifying subjective information. However, the meaning and relevance of the generated topics must be deduced, and therefore, some qualitative aspects remain. Topic modeling has been used to evaluate depression, anxiety, and other symptoms from public comments in social media posts [16,17]. Importantly, public online comments have also been used to detect early signs of cognitive decline [18]. We sought to evaluate a similar approach for investigating CRCI given the advantages that it affords, including access to a large data set that is without cost, representation of a wide variety of individuals, and reduced bias related to assessment context [16].

Traditional qualitative content analysis is used to describe phenomena and generate evidence for larger quantitative descriptive studies or for theory generation [19]. Researchers code narrative data to determine the existence and frequency of concepts within the text. This is an inductive process as broad categories are generalized from the specific content that is identified in the data. Qualitative content analysis has been employed in a few prior studies of CRCI [9,20-24], but studies employing quantitative methods are far more common. Further, previous qualitative studies were not used to guide development of CRCI assessments. We aimed to demonstrate how qualitative themes can provide novel insights regarding patient experiences with CRCI that could potentially inform the development of cancer-specific assessments in the future.

## Methods

### Topic Modeling

We identified 10 public online forums by conducting internet searches with the terms *chemobrain*; *cancer*; *cognition*; *survivorship*; and *supportive care*. These forums consisted largely of group discussions/conversations regarding cancer survivorship–related topics but also included responses to online articles about *chemobrain*. We extracted comments using automated data scraping functions in the R Statistical Package version 4.0.2 (R Foundation). Comments were cleaned (removed contractions, symbols, links, stop words [eg, *the, has, this*]) and converted to a document-term matrix for topic modeling, again using automated functions in R. Latent topics were discovered using latent Dirichlet allocation [25] with Gibbs sampling. This yielded a probability that a forum comment belonged to a particular latent topic. Topics, in this context, are groups of words that are related to each other. There is no standard for determining the optimal number of topics to look for. Therefore, we examined the rate of perplexity change across a range of topic values to estimate the optimal number of topics [26]. Latent Dirichlet allocation was conducted in R using the *topicmodels* library.

### Content Analysis

The forum responses were aggregated into a single transcript and reviewed independently by 2 of the coauthors (AMH and WT) using an inductive qualitative content analysis approach [19,27,28]. This approach allows for the distillation of words into fewer content-related categories, which is done manually [28]. Each coauthor read through the entire transcript at least once to become familiar with the data, then initiated the line-by-line coding process. The units of analysis were words and phrases. Codes were inductively grouped into larger categories that emerged directly from the data, without an organizing framework, noting quotes from participants illustrating the categories [28]. The coauthors then met to compare and collapse categories and complete the abstraction process. Abstraction involves forming general descriptions and meanings of the final categories [28]. AMH and WT were kept blinded to the topic modeling results so that their findings would not be influenced by the topic modeling.

### Ethics Approval

This study utilized public data that do not require institutional review board approval.

## Results

### Overview

We identified 145 online forum comments. Comments were posted by single online usernames, and all included first-person pronouns (eg *I*, *my*). Thus, comments were assumed to represent 145 individuals. Comments had a mean word count of 146 (SD 65).

### Topic Modeling

**Figure 1:**
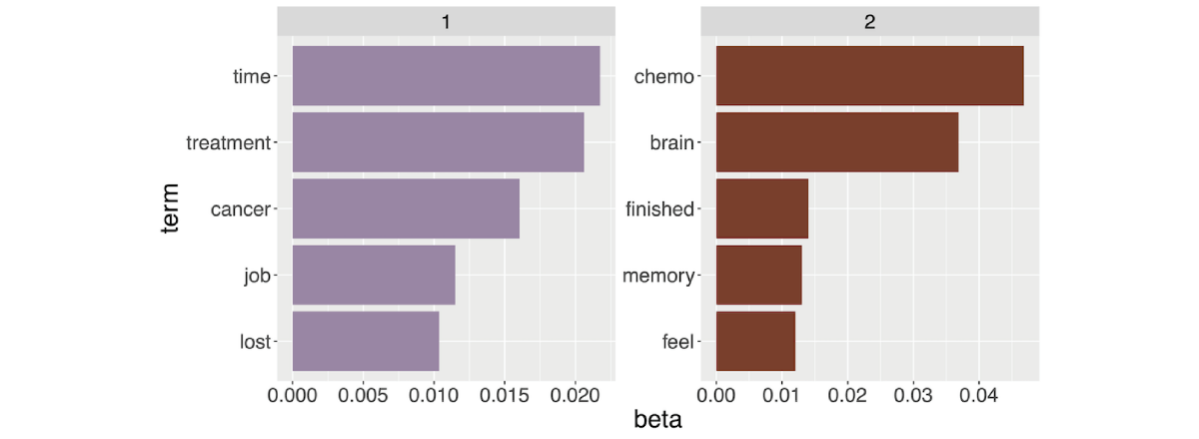
Topic modeling of free-text comments. Latent Dirichlet allocation (LDA) analysis of online comments identified 2 topics related to cognitive effects of cancer and its treatments. Beta = probability that the term belongs to that topic. We interpreted topic 1 as reflecting external factors and topic 2 as indicating internal factors leading us to hypothesize regarding potential contribution of locus of control to subjective cognitive impairment. Figure created using ggplot in the R Statistical Package.

As shown in Figure 1, topic modeling identified 2 latent topics that we qualitatively interpreted, by consensus, as representing *external* (topic 1) and *internal* (topic 2) factors related to individuals’ concerns about cognitive functioning. These findings lead us to hypothesize regarding the potential contribution of locus of control to CRCI.

### Content Analysis

#### Major Categories

The following major categories were identified: *symptoms*, *emotional/psychological impacts*, *coping*, *“chemobrain” is real*, *change over time*, and *function*.

#### Symptoms

The online comments largely described cognitive symptoms along with related physical symptoms (eg, fatigue, neuropathy). The most common cognitive symptom discussed was impaired memory, specifically trouble with short-term memory or remembering things “on the fly.” One person described, a “Total inability to cope with remembering things” and another said, “My mind couldn’t remember things that used to be easy for me.” Other comments frequently mentioned word-finding problems, difficulty concentrating, and a slowness or lack of mental sharpness/speed. People also suggested explanations other than chemotherapy for their symptoms such as other cancer treatments (eg, tamoxifen, radiation), having too much on their plates (ie, information overload), getting older, or developing dementia.

#### Emotional/Psychological Impact

Throughout the online comments, many people discussed the strong emotional and psychological impacts of their cognitive symptoms and changes. These were sometimes described in the context of feeling worried, upset, anxious, or scared of their cognitive symptoms. Other times symptoms were described in the context of extreme frustration, feelings of anger, and being overly stressed. One person said, “It’s a total frustration” and another said, “I am really suffering.” Additionally, many described feelings of embarrassment, loss of confidence or self-reliance, or even feeling nervous about their own cognitive performance. One person illustrated this point by saying, “Cancer and memory loss can corrode my intellectual self-esteem and only compound the problem.”

#### Coping

Many people referenced ways of coping with their cognitive changes by engaging in brain-healthy behaviors such as exercise, stress reduction, or puzzles. Others referenced using different medications such as Ritalin, or supplements (eg, CoQ10) to improve their cognitive stamina and function. Others talked about utilizing compensatory strategies for better functioning in their everyday lives such as making lists, using smartphone capabilities, slowing down, and planning more.

#### “Chemobrain” Is Real

Most of the online comments related to the idea of validating that chemobrain, or cognitive changes related to cancer and cancer treatment, are very real. Different words were used to describe the phenomenon such as “chemo haze,” “chemo fog,” a “scrambled brain,” and “brain is total mush.” Some people voiced frustration with lack of awareness or validation from their providers and noted that, “it would have helped if there had been more awareness [about chemobrain]”. Similarly, people made sure to emphasize that those suffering from chemobrain are not alone. For example, one person said, “Don’t feel you have to cope with this on your own” and another said, “we are with you”.

#### Change Over Time

A common theme that emerged from the online comments was the experience of cognitive function changing over time. For instance, many people described ongoing cognitive difficulties since the end of their treatments and in some cases declining or getting worse over time. By contrast, others described improvements in cognitive difficulties since their treatments ended, saying they are “doing better with time”. Others described cognitive symptoms as getting worse throughout individual days, with better functioning in the morning and dysfunction in the evenings or when they were tired.

#### Function

Finally, many of the online comments centered around the theme of functioning in their everyday lives—from social and interpersonal interactions to occupational performance and in many cases debilitation, or lack of function. One person said, “I can sit and listen to someone talk and then it’s like I feel thick, like I just don’t understand what’s being said.” Others talked about slow returns to work, workload reduction, and lack of ability to do the work they did prior to their cancer. Several people described losing their jobs due to their cognitive problems and inability to function at previous levels. Some talked about an inability to do the things they wanted to in their lives, or a loss of the person they were prior to their treatments. One person said, “I wish I could be my old self.”

## Discussion

### Principal Findings

We evaluated public online comments regarding CRCI for qualitative themes using both topic modeling and content analysis. The goal of this study was to demonstrate how qualitative themes can provide novel insights regarding patient experiences with CRCI that could potentially inform the development of cancer-specific self-report assessments. Topic modeling identified 2 topics from online comments that we interpreted as representing “internal” and “external” factors related to CRCI. Taken together, these topics suggested the potential importance of locus of control when considering CRCI symptoms.

A previous qualitative study regarding CRCI also identified perceived control as a major theme derived from interviews with 12 participants [29]. Locus of control regarding health has been shown to be important for cancer survivorship [30] and quality of life [31], and is correlated with self-management behaviors [32,33]. Importantly, locus of control has been shown to be a modifiable factor in cognitive function [34]. One study found a correlation between internal locus of control and self-reported cognitive function in patients with colorectal cancer [35]. Internal locus of control is associated with adaptive coping [36], likely because it engenders a greater sense of agency and mastery over one’s situation. However, no studies have examined the relationship between locus of control, coping mechanisms, and cognitive impairment in patients with cancer.

A total of 6 categories were identified using content analysis including symptoms, emotional/psychological impacts, coping, “chemobrain” is real, change over time, and function. Our findings support themes identified in previous qualitative studies of CRCI such as cognitive symptoms, negative emotional reactions to cognitive changes, major negative effects on quality of life, trying different coping strategies, and a need for validation [9,20-24]. However, our sample was much larger as most previous qualitative studies have been conducted with samples of 10-25 participants. The largest study to date involved a total of 74 breast cancer survivors [9]. Our study findings summarize experiences of approximately 145 individuals, representing the largest qualitative study on *chemobrain* to our knowledge. Second, content analysis is typically used to analyze data collected through individual interviews or small focus groups. Our use of content analysis to evaluate a large volume of public comments is novel.

Our results indicated that each text analysis method provides unique information and insights. Topic modeling indicated 2 topics, or categories, while content analysis indicated 6 categories. While the number of categories is different, there was conceptual overlap in the categories. For example, the content analysis categories *symptoms*, *emotional/psychological impacts*, and *coping* could align with the *internal* topic, while *“chemobrain” is real*, *change over time*, and *functioning* could align with the *external* topic. In fact, both techniques pointed to the importance of coping mechanisms as a significant theme.

As content analysis is used to develop an understanding of the meaning of the intentions, consequences, and contexts of the words [19,27,28], the findings are inherently richer and can be considered more “macroscopic” than topic modeling, which focuses on the more “microscopic,” word level of narratives. Topic modeling allowed for latent analysis of the forum comments, which the content analysis did not. The locus of control theme suggested by topic modeling was not readily apparent from the online comments and thus this technique provided increased depth of understanding.

Based on our findings, it would be important to include questions regarding coping mechanisms and locus of control when assessing patients for CRCI. The ways in which patients must adjust their approach to cognitive demands in real-world situations may be a more sensitive measure of their cognitive status than performance on a cognitive test. In fact, CRCI was historically controversial due to normal performance on cognitive tests by patients reporting cognitive deficits [12]. Some have suggested that patients are able to compensate for or adapt to cognitive effects, masking the underlying deficit [37]. However, compensation is effortful and the lengths that one must go to adapt would be reflective of the masked deficit. Currently, there are no standardized self-report measures for CRCI that include evaluation of coping mechanisms. However, the Compensatory Cognitive Strategies Scale was developed and validated in persons with multiple sclerosis to measure the frequency of using 24 cognitive strategies [38]. This scale could easily be adapted and validated for use in cancer populations.

We would expect that patients with internal locus of control would have greater tendency to utilize compensatory strategies when dealing with cognitive effects. A focus on locus of control could also have implications for treating CRCI partly by changing individual attributions regarding cognitive failures [39]. Mindfulness-based interventions for CRCI [40] may work via locus of control by exploring the way one thinks about successes and failures. We previously suggested that cognitive training may operate in part by increasing locus of control [41]. Currently, there are no standardized self-report measures for CRCI that include evaluation of locus of control. However, there are several existing locus of control measures, including within the public domain, that could be used or adapted for the evaluation of CRCI (eg, [42])

### Limitations

The reliability of topic modeling is affected by the sizes of the corpus and its individual documents. While there are no set benchmarks for these, larger samples are typically better for distinguishing topics, and therefore, we may have lacked the ability to find additional latent topics. Even though content analysis is commonly used in health sciences research to characterize phenomena and generate theories, individual interpretations can influence or bias the results of content analysis [19]. The interpretation of latent topics is similarly subjective.

### Conclusions

Our results suggest that analysis of free-text narratives may provide unique insights regarding subjective experience of cognitive function that could guide development of new CRCI assessments. Although this is not the first study to reveal important qualitative themes related to CRCI, little has been done thus far in terms of incorporating these themes into actual CRCI assessments. This may be due in part to the inherent difficulty in acquiring large samples of data from traditional qualitative methods or a lack of qualitative researchers invested in this field. Applying topic modeling would also be advantageous in terms of increased analytical efficiency given that it is largely automated. Although some advanced computational and computer science expertise is often required for such analyses, many user-friendly resources are currently available, such as Amazon Comprehend (Amazon Web Services, Inc.), MonkeyLearn (MonkeyLearn, Inc.), RapidMiner (RapidMiner, Inc.), and Google Cloud Natural Language (Google, Inc.), which require little if any expertise.

## Abbreviations

CRCI: cancer-related cognitive impairment
